# Predictors of Access to Healthcare: What Matters to Rural Appalachians?

**DOI:** 10.5539/gjhs.v4n6p23

**Published:** 2012-08-16

**Authors:** Susan L. Wilson, Cynthia Kratzke, Jill Hoxmeier

**Affiliations:** 1 Department of Public Health Sciences, New Mexico State University, Las Cruces, NM, USA

**Keywords:** health disparities, access to healthcare, perceptions, behavioral model, rural health

## Abstract

**Objective:**

Lack of access to healthcare is frequently cited as a primary reason for health disparities globally, especially in poor, rural areas such as Appalachia in the U.S. This study examined predictors of perceived access to healthcare among residents in a poor, medically underserved, rural Appalachian community.

**Methods:**

The study was guided by the revised behavioral model of healthcare services utilization. Self-reported survey data were obtained from a convenience sample of 921 residents in rural Tennessee.

**Results:**

The majority of respondents in this study did not perceive access to healthcare to be a problem in their community. Financial factors, health status, and associated social factors negatively affected only a small number respondents’ perceptions of access to healthcare.

**Conclusions:**

Despite the presence of multiple factors previously shown to affect access to healthcare, the majority of respondents in this study did not perceive access to healthcare to be a problem in their community. Results of this study suggest that to understand an individual’s passage through the healthcare system, the contextual aspects of healthcare utilization, should be added to coverage, services, timeliness, and workforce as a fifth component of access to healthcare. Assessing perceived need and associated cultural factors that affect individuals’ concepts of health and wellness represent important areas for future exploration to explain observed health disparities. Additionally, findings showed that having sufficient quality and quantity of healthcare professionals and services in a community or region may be necessary, but not sufficient to explain health disparities and the underlying reasons why individuals choose or choose not to seek health services.

## 1. Introduction

Lack of access to healthcare is frequently cited as a primary reason for health disparities globally, and especially in poor, rural areas such as Appalachia in the U.S. Glaring inequalities in health and access to healthcare exist both between and within countries (Marmot, Friel, Bell, Houweling, & Taylor, 2008). Furthermore, there is approximately a 20-year gap in life expectancy between the least and most advantaged groups in the U.S. (Murray et al., 2006). The causes, meanings, and implications of access to healthcare have been identified and analyzed from multiple perspectives for decades (Institute of Medicine, 1993; Ross, 2002). This study examined factors that influence perceptions of access to healthcare among residents in a poor, medically underserved, rural Appalachian community.

“*Access*” to healthcare is a broadly based conceptual term used to describe a group’s or an individual’s ability to obtain needed medical services (Institute of Medicine, 1993). A common assumption is that access to healthcare equates with both financial and systemic resources in an area (Aday & Andersen, 1974). According to Healthy People 2020, access to healthcare services “encompasses four components: coverage, services, timeliness, and workforce” (U.S. Department of Health and Human Services (HHS), 2010, para. 4). Among the factors that act as barriers to health services are transportation, rurality, lack of financial resources, lack of health insurance, and language (Committee on Monitoring Access to Personal Health Care Services-Institute of Medicine, 1993).

Historically, arguments regarding the need for better access to healthcare range between two extremes. At one end, healthcare as a right, i.e., proponents assert that access to quality healthcare is a human right stressing that all individuals should be able to acquire an adequate level of care without excessive burden (President’s Commission for the Study of Ethical Problems in Medicine and Biomedical and Behavioral Research, 1983; Reed, 1952). At the other end of the spectrum, proponents argue that a person’s health status relies principally on individual choices. Positions at either extreme, as well as all positions in between, affect the distribution of health disparities and policy decisions designed to eliminate the disparities. Either approach implicitly assumes that these factors affect the quality and quantity of an individual’s passage through the healthcare system. However, few studies showed that access to healthcare, utilization patterns, race, and income alone explain differences in life expectancy among Americans (Murray et al., 2006).

While depressed economics and limited access to health services do not cause uniform ill-health among rural communities throughout the nation, rural Appalachians have had some of the highest poverty rates and lowest health indicators of any U.S. residents (Murray et al., 2006). Forty-two percent of all Appalachians live in rural communities with less than 2,500 persons and the proportion of older adults is higher as compared to the rest of the country (Goins, Williams, Carter, Spencer, & Solovieva, 2005; Haaga, 2004). Appalachia, as defined by the US Congress, is a geographic region encompassing 205,000 square miles in 410 counties in 13 states and ranges from northeastern Mississippi to southern New York (Pollard & Jacobsen, 2012). The area’s rugged topography and isolated valleys result in low population density and less travelled areas, which may affect access to and utilization of healthcare (Arcury et al., 2005; Pollard, 2003).

The Appalachian region of Tennessee (52 counties) has a population density of 136.5 inhabitants per square mile as compared with 165.3 for the rest of the state and 122.3 for the region as a whole (Pollard, 2003). Tennessee was one of the lowest ranked states in the U.S. for health, ranking 47^th^ in 2006, having moved up one position from 48^th^ in 2005 (United Health Foundation, 2006). Hawkins County, Tennessee [site of this study], was rated 27^th^ out of 95 counties in overall health rankings in Tennessee for 2006 and dropped to 34^th^ in 2012 (Population Health Institute, 2012; Tennessee Institute of Public Health, 2006). Regarding health determinants, the county ranked 62^nd^ overall; 61^st^ in healthcare; 73^rd^ in health behavior; 54^th^ in socioeconomic factors; and 45^th^ in physical environment (Tennessee Institute of Public Health, 2006).

## 2. Materials and Methods

### 2.1 Study Design

This study was guided by the revised behavioral model of healthcare services utilization (Andersen, 1995). The behavioral model has been revised numerous times with each successive iteration seeking to better understand utilization of healthcare services for prevention, care-seeking, and recovery activities (Aday & Andersen, 1998; Andersen, 1995; Andersen & Davidson, 2001; Andersen et al., 2002; Borders, Aday, & Xu, 2004; Phillips, Morrison, Andersen, & Aday, 1998). The revised behavioral model addresses environmental, population, health behavior, and outcomes characteristics (Andersen, 1995). Population characteristics that influence personal health practices and use of health services fall into three categories: predisposing, enabling, and need factors. Predisposing factors may include age, gender, ethnicity, and health beliefs. Example enabling factors include resources such as income, education, insurance or medical care organizations. Need factors may include, for example, evaluated or perceived health status and issues of consumer satisfaction.

### 2.2 Data Source

Data for this project were secondary data from a cross-sectional study implemented by the Tennessee Department of Health, Northeast Regional Health Office (TDH-NRHO). The purpose was to obtain additional information for use in county health planning and development efforts.

#### 2.2.1 Sample

A convenience sample of adults living in Hawkins County, located in the northeast Tennessee region, was surveyed during the spring 2006. The county’s estimated population was 56,196 residents in 2005, a population that had increased 4.9% since 2000. The population aged 65 years and older made-up 14.3% of the county’s population. The population of Hawkins County was 97.4% white, higher than the state average of 80.7% white (U.S. Census Bureau, 2007). Males account for 49% or the population, while females are 51% of the population (U.S. Census Bureau, 2012).

### 2.3 Data Collection

Data collection was completed using a self-administered, paper and pencil survey instrument (“Northeast Tennessee Regional Health Assessment”) developed by the Tennessee Department of Health Northeast Regional Office in Johnson City. The survey content was designed by TDH-NRHO staff to complement information acquired by the Behavioral Risk Factor Surveillance System (BRFSS), a state-based, randomized telephone interview conducted by the state in conjunction with the Centers of Disease Control and Prevention (CDC). Detailed questions were included to address specific concerns arising from the region and questions were patterned after BRFSS surveys.

Respondents were asked to complete a 67-item survey covering demographics, healthcare services utilization, and perceived personal/community health care needs. Surveys were delivered to respondents, usually in their place of work or in public venues, such as a physician’s office. Once the survey was completed, it was returned to the researcher who initially hand-delivered the survey.

Participants were able to stop the survey at any time during the process and were able to skip questions if they chose. As a result, a relatively high number of individuals did not report information for all questions on the survey, including basic demographic information. Although deleting observations produces potential loss of information, this method of accommodating missing values was adopted for this paper. Therefore, results for statistical tests are reported using completed numbers for each test.

### 2.4 Dependent Variable

The dependent variable (DV) was a dichotomous measure of the degree to which individuals in the study rate perceived access to healthcare to be a problem in their community. Responses of severe or moderate problems were grouped into one analytical category, “problem”. Responses that indicated access to healthcare was not a problem or did not know if there was a problem were grouped as “not a problem”.

### 2.5 Independent Variables

The following independent variables were selected pursuant to Aday and Andersen’s model (1998).

#### 2.5.1 Predisposing Factors

Three predisposing demographic factors were analyzed: gender (female, male); three age cohorts (18-39, 40-59, and 60+ years); and, race/ethnicity (white, non-white). Access problems would perhaps be expected to be higher among younger members with children and older members with limited mobility.

#### 2.5.2 Enabling Factors

Several factors that represent the availability of sociocultural (non-material) and financial (material) resources were examined. Enabling factors were reported in four categories: social capital, human capital, material capital, and healthcare resources. Social capital is a nonmaterial resource, which indicates interpersonal ties. Two social capital factors were analyzed: marital status (married, not-married) and length of residency in the county (<20 years, 20+ years). Education is a measure of human capital, which is a hypothetical measure of one’s ability to maximize productive potential (Borders et al., 2004). Education is categorized as the highest level of education completed (elementary/grammar school; high school graduate; college graduate or more). A measure of potential ability to contribute financially to healthcare was measured by four factors: income and presence or absence of health insurance, Medicaid, and a doctor. Household income per year was broken down into poor (<100% of poverty for 2006 (U.S. Department of Health and Human Services, 2006) - <$19,999); low income (100 -150% of poverty, $20,000 – $29,999); middle income (150 – 300% of poverty, $30,000- $59,999); and, high income (>300% of poverty, ≥$60,000). Insurance data were not identified by source, with the exception of Medicaid (TennCare), which was a separate question. A person could indicate that they had both insurance and Medicaid; therefore, these indicators are not independent of one another. Medicare among the elderly and disabled was included in the insurance measure. Respondents were also asked identify whether or not they had a doctor. Each of these factors were be hypothesized to facilitate access to healthcare.

#### 2.5.3 Need Factors

Four indicators of health related quality of life needs were assessed in relation to access to healthcare. Respondents were asked to identify “to what degree have the following affected you or someone in your family in the past 12 months?” for the following situations: “Didn’t have enough money for healthcare (doctor, dentist, prescriptions)” ($$Healthcare), “Didn’t have enough money for insurance” ($$Insurance), “Unable to get in-home care or adult daycare for an elderly, disabled, or ill person” (In-Home Care), and “Unable to get transportation for medical, dental, or mental health appointments” (Transportation). As well as representing need among the population, problems with these factors might be viewed as potential barriers to healthcare utilization.

Seven self-reported health conditions were assessed based presence or absence in the respondent or a family member experienced during the previous 12 months. The seven conditions include: cancer, diabetes, heart attack, high blood pressure, high cholesterol, respiratory problems, and stroke. Each condition is identified by the CDC as among the most prevalent, costly, and preventable of health problems (Centers for Disease Control and Prevention, 2008).

### 2.6 Statistical Analysis

Data analysis was conducted using descriptive statistics, frequency distributions, Chi-square (χ^2^) analysis, and binary logistic regression analysis. Only factors found to be significant in Chi-square analysis were included in the logistic regression analysis table. Data analysis was conducted using SPSS Version 19 statistical software. A *p* value of <0.05 was used for statistical significance.

## 3. Results

### 3.1 Descriptive Analysis

Descriptive statistics for the sample (N=921) are shown in Table 1. The majority of participants were female (83.3%), white (97.5%), over 40 years of age (71.4%), and had an education of high school or less (58.9%). No statistical significance was identified for race/ethnicity. Therefore, race/ethnicity was not analyzed in remaining evaluations due to the high proportion of whites in the sample and in the county population. Most participants were married (72.4%) and had lived in the county for over 20 years (69.9%), indicating a demographically stable population. Poor and low income respondents (<$30,000 annual household income) constituted 38.9% of the sample. Most respondents had public or private medical insurance (90.8%), most were not on Medicaid (88.1%), and almost all respondents had a doctor (94.6%). A majority of individuals did not find the health related quality of life factors to be a problem. Respondents who stated these issues were not a problem included 58.3% for getting healthcare from a doctor, dentist, or pharmacist; 60.5% for getting insurance; 78.8% for getting in-home or daycare services; and, 82.0% for transportation to healthcare services. The most frequently reported health condition among respondents was high blood pressure (67.0%). Less than half of respondents reported cancer (41.3%), diabetes (43.8%), heart attack (33.9%), high cholesterol (47.1%), respiratory problems (24.3%) or stroke (19.3%) in the past 12 months.

**Table 1:** Descriptive statistics of predisposing, enabling, and need characteristics of participants

	N	(%)
**Predisposing Factors**		
Gender	761	100.0
Female	634	83.3
Male	127	16.7
Age Group	873	100.0
18-39 years	250	28.6
40-59 years	368	42.2
60 years or more	255	29.2
Race/Ethnicity	917	100.0
White, non-Hispanic	894	97.5
Hispanic, Latino/a	4	0.4
Native American, Alaskan Native, Native Hawaiian, Pacific Islander (NA/AN/NH/PI)	13	1.4
Black, non-Hispanic	6	0.7

**Enabling Factors**		
*Enabling - Social Capital*		
Marital Status	917	100.0
Married	664	72.4
Not married	253	27.6
Length of Residence in County	918	100.0
<20 years	276	30.1
20 or more years	642	69.9
*Enabling - Human Capital*		
Education – highest level achieved	914	100.0
Elementary/Grade School	85	9.3
High School	453	49.6
College or more	376	41.1
*Enabling - Material Capital*		
Household income	862	100.0
Poor, <$19,999	215	25.0
Low income, $20,000 - $29,999	121	14.0
Middle Income, $30,000 - $59,999	213	24.7
Upper Income, ≥$60,000	313	36.3
*Enabling – healthcare resources*		
Do you have health insurance?	900	100.0
Yes	817	90.8
No	83	9.2
Are you on TennCare (Medicaid)?	890	100.0
Yes	106	11.9
No	784	88.1
Do you have a doctor?	892	100.0
Yes	844	94.6
No	48	5.4

**Need Factors**		
*Need factors -- Health Related Quality of Life*		
Need $$ for Healthcare	859	100.0
Problem	358	41.7
Not a Problem	501	58.3
Need $$ for Insurance	863	100.0
Problem	306	35.5
Not a Problem	557	64.5
Need assistance with In-home care	837	100.0
Problem	111	13.3
Not a Problem	726	86.7
Need Transportation	849	100.0
Problem	94	11.1
Not a Problem	755	88.9
*Need factors - Self-reported health conditions*		
Cancer	921	100.0
Yes	380	41.3
No	541	58.7
Diabetes	921	100.0
Yes	403	43.8
No	518	56.2
Heart Attack	921	100.0
Yes	312	33.9
No	609	66.1
High Blood Pressure	921	100.0
Yes	617	67.0
No	304	33.0
High Cholesterol	921	100.0
Yes	434	47.1
No	487	52.9
Respiratory problems	921	100.0
Yes	227	24.6
No	694	75.4
Stroke	921	100.0
Yes	178	19.3
No	743	80.7

### 3.2 Bivariate Analysis

Table 2 shows results of bivariate analysis of perceived access to care problems and each of the predisposing, enabling, and need factors. A statistically significant response of “no problem” was found for most variables. Perceived access to healthcare was found to be a “problem” only among the most vulnerable in the population and was associated with gender (female), age (young), education (low), income (poor), lack of health insurance, not having a doctor, each of the financial need factors, and respiratory problems. Marital status, length of residency in the county, or being a Medicaid recipient were not statistically significant indicators of perceived access to healthcare problems. Having cancer, diabetes, heart attack, high blood pressure, high cholesterol or stroke were not statistically significant indicators of perceived healthcare access.

**Table 2:** Sample characteristics by perceived access

	Total		Perceived Access Problem		Perceived Access Not a Problem		*p* Value (Pearson χ^2^ Test Result)

	N	Percent	N	Percent	N	Percent	
**Predisposing Factors**							
Sex							.025
Female	606	83.4	303	50.0	303	50.0	
Male	121	16.6	47	38.8	74	61.2	
Age (category)							<0.001
18-39 years	249	28.9	129	51.8	120	48.2	
40-59 years	357	41.5	190	53.2	167	46.8	
60+ years	255	29.6	76	29.8	179	70.2	

**Enabling Factors**							
*Social Capital*							
Marital Status							0.297
Married	638	73.2	298	46.7	340	53.3	
Non married	234	26.8	100	42.7	134	57.3	
Residency in County							0.659
< 20 years	264	30.2	118	44.7	146	55.3	
20 years or more	611	69.8	283	46.3	328	53.7	
*Human Capital*							
Education							<0.001
Elementary/Grammar School	69	7.9	19	27.5	50	72.5	
High School graduate	432	49.7	172	39.8	260	60.2	
College or more	369	42.4	209	56.6	160	43.4	
*Material Capital*							
Income							0.003
Poor	193	23.4	82	42.5	111	57.5	
Low income	115	14.0	46	40.0	69	60.0	
Middle income	208	25.2	82	39.4	126	60.6	
Upper income	308	37.4	166	53.9	142	46.1	
*Healthcare Resources*							
Health Insurance							<0.001
Yes	789	91.2	348	44.1	441	55.9	
No	76	8.8	50	65.8	26	34.2	
Medicaid							0.674
Yes	100	11.7	48	48.0	52	52.0	
No	756	88.3	346	45.8	410	54.2	
Doctor							0.025
Yes	813	94.8	367	45.1	446	54.9	
No	45	5.2	395	46.0	463	54.0	

**Need Factors**							
*Health Related Quality of Life*							
Need $$ for Healthcare							<0.001
Problem	342	41.1	215	62.9	127	37.1	
Not a Problem	490	58.9	175	35.7	315	64.3	
Need $$ for Insurance							<0.001
Problem	293	35.0	183	62.5	110	37.5	
Not a Problem	546	65.0	208	38.3	335	61.7	
Unable to get In-home Care							.002
Problem	107	13.1	65	60.7	42	39.3	
Not a Problem	708	86.9	316	44.6	392	55.4	
Unable to get health related transportation							<0.001
Problem	90	10.9	64	71.1	26	28.9	
Not a Problem	737	89.1	389	47.0	438	53.0	
*Self-reported health conditions*							
Cancer							0.403
Yes	365	41.7	161	44.1	204	55.9	
No	511	58.3	240	47.0	271	53.0	
Diabetes							0.136
Yes	389	44.4	189	48.6	200	51.4	
No	487	55.6	212	43.5	275	56.5	
Heart Attack							0.643
Yes	302	34.5	135	44.7	167	55.3	
No	574	65.5	266	46.3	308	53.7	
High Blood Pressure							0.223
Yes	600	68.5	283	47.2	317	52.8	
No	276	31.5	118	42.8	158	57.2	
High Cholesterol							0.853
Yes	423	48.3	195	46.1	228	53.9	
No	453	51.7	206	45.5	247	54.5	
Respiratory Problems							0.016
Yes	224	25.6	118	52.7	106	47.3	
No	652	74.4	283	43.4	369	56.6	
Stroke							0.250
Yes	171	19.5	85	49.7	86	50.3	
No	705	80.5	316	44.8	389	55.2	

### 3.3 Multivariate Analysis

Logistic regression analysis was conducted on variables found to be statistically significant in bivariate analysis to identify factors that influence access to healthcare perceptions (Table 3). Healthcare access was categorized into either “Problem” or “Not a Problem” for this analysis. A test of the full model against a constant only model was statistically significant, indicating that the predictors as a set reliably distinguished between those who thought access to health was a problem and those who thought access to care was not a problem(chi square = 103.983, p< 0.001 with df = 11). Gender (p = 0.048), education (<0.001), income (p = 0.01), having insurance (p = 0.001), healthcare dollars (p = 0.001) and ability to get medical transportation (p = 0.005) made significant contributions to the prediction model. Age, having a doctor, having sufficient funds for insurance, being unable to get in-home care, and having respiratory problems were not predictive factors in the model.

**Table 3:** Probability of perceiving an access to healthcare problem binary logistic regression results

Variable	Coefficient	Standard Error	*P* Value	Odds Ratio	95% Odds Ratio Confidence Interval
Gender	-0.487	0.247	0.048	0.615	0.38-1.00
Age	0.127	.0132	0.333	1.136	0.89-1.47
Education	-.0729	0.186	<0.001	0.482	0.36-0.69
Income	-0.253	0.099	0.011	0.777	0.64-0.94
Insurance	-1.316	0.396	0.001	0.268	0.12-0.58
Doctor	-.786	0.488	0.107	0.456	0.76-1.19
Healthcare $$	0.864	0.259	0.001	2.374	1.43-3.94
Insurance $$	0.269	0.276	0.330	1.309	0.76-2.25
In-home $$	-0.186	0.299	0.534	0.930	0.46-1.49
Transportation $$	0.982	0.351	0.005	2.669	1.34-5.31
Respiratory Problems	-0.326	0.206	0.113	0.772	0.48-1.08

## 4. Discussion

This study used a population based survey to assess perceived access to healthcare among rural Appalachians in one Tennessee County. Generalizability of the results is limited by the use of a cross-sectional, convenience sample. Due to differences from county means in household income (the study group was poorer), educational level, and gender proportions, the results may not be representative of the whole county population or Appalachia as a whole. Also, the design of the study may generalizability because it is not possible to differentiate private from public health insurance among respondents.

Other studies have used variations of the behavioral health model originally developed by Andersen in the 1960s and modified numerous times by various researchers (Aday, 2001; Aday & Andersen, 1998, Andersen, 1995; Borders, Aday, & Xu, 2004). This study sought to use the general framework of the behavioral health model to determine if the population perceived their community to have equitable access to healthcare. As noted by Andersen (1995, p.4), “Equity is in the eyes of the beholder.” The research began with the assumption that as perceived access to care increased, barriers to access decreased as measured in predisposing, enabling, and need factors identified within the population. In reviewing the framework for health services research identified by Aday (2001), this research attempted to bridge predisposing, enabling, and need factors with with physical, social, and economic factors in the health services environment. This study added the factor of perceived access to healthcare (a sociocultural factor) to the overall behavioral health model. Perceived access was analyzed as the dependent variable to determine which, if any, of the predisposing, enabling, and need factors affect this potential access measure in the community. To the best of the authors knowledge, this is the first article to analyze perceived access to healthcare in this capacity.

The study shows important findings for understanding perceived access to healthcare and offers potentially new information for health policy and community planning efforts. Previous studies have documented numerous characteristics of health behavior among rural Appalachians (Huttlinger, Schaller Ayers, & Lawson, 2004; Wilson, Huttlinger, & Reddy, 2010).

A clearer picture of “who” perceived healthcare access to be a problem is demonstrated in the multivariate analysis. Only a few characteristics measured contributed statistically to the probability that predicting access to healthcare was a problem: one pre-disposing factor (gender); three enabling factors (insurance, education and income); and two need factors (financial difficulties paying for health care and difficulty with transportation for medical care).

Two predisposing factors were statistically significant when bivariate, Chi-square analysis was conducted; however, age was not a significant predictor of perceiving a problem with healthcare access in multivariate analysis. Gender, a pre-disposing factor, was significant but not highly so in predicting perceived access problems. In the study, females were evenly divided between access being a problem and access not being a problem, but males were more inclined to perceive that access was not a problem. This phenomenon may be due to the proportionally smaller number of males in the sample than females.

Previous studies have shown that three main enabling factors are often associated with health seeking behaviors and realized access to healthcare: poverty, being uninsured, and not having a regular doctor. Having a customary source of regular healthcare usually results in more health seeking behavior, thus greater access to care (Hayward, Bernard, Freeman, & Corey, 1991; Lambrew, Defriese, Carey, Ricketts, & Biddle, 1996; Sox, Swartz, Burstin, & Brennan, 1998). Although this study did not measure doctor visits per year, it did show that most respondents report having a doctor. Findings further showed that having a regular healthcare provider was not significantly associated with perceived access to care. This is true also when measured against the prevalence of major chronic diseases that require specialist care, e.g., cancer or heart conditions.

From a planning perspective, Hawkins County, Tennessee, still falls short of needed healthcare providers. The county is a designated Health Professional Shortage Area (HPSA) for primary medical care, dental, and mental health services and is designated as a whole county Medically Underserved Area (MUA) (Health Resources and Services Administration, 2007). Findings suggest that while the county objectively has an access to healthcare problem; subjectively, most individuals did not perceive a problem existed with access to healthcare in their community. It should be noted that residents have specialty and tertiary services available within less than 100 miles, a fact that may affect perceived access to care. Regional services may be sufficiently available to contextually function as locally accessible to residents from their individual perspectives. Paradoxically, Huttlinger et al. (2004) reported that having a high primary care provider to population ratio may result in greater access, but does not result in better health status in one rural Appalachian population. Based on this study’s findings, it may be that assessing perceived access could clarify some of the discrepancies seen in other studies.

Insurance has also been demonstrated to affect realized access to healthcare. For example, Hafner-Eaton (1993) reported that non-elderly insured individuals were consistently more likely to have received healthcare than the uninsured, thus indicating that insurance is a key enabling factor affecting realized access to care. Spillman (1992) found that increased insurance coverage would increase access to healthcare and, thus, increase utilization. Although measures of actual utilization were not undertaken, this study paradoxically showed that having insurance was a statistically significant predictor of perceived access, i.e., most respondents (54%) did not perceive an access problem. It is, however, important to note that a higher percentage of uninsured individuals perceived a community access to healthcare problem than did others in the study. This finding suggests that perceived access differentially affects the uninsured, even though access was overall thought not to be a problem.

Poverty and material capital have been shown to be positively associated with health in a number of studies (Borders, Aday & Xu, 2004; Moss, 2000; Veenstra, 2000, 2002). Regarding poverty as a predictor of a perceived access problem, it is important to note that education and income were significantly related to one another in the study population (Pearson’s Chi-square = 210.982, df = 4, p < 0.001), indicating that income and education were mutually dependent so they will be discussed as one entity. Median household income in Hawkins County (2006-2010) was $35,392, with 18.3% of the population below the poverty level (U.S. Census Bureau, 2012). In the study group, 37.4% fall roughly at or below the median income level for the county, with 23.4% of those below the poverty level. Based on previous studies, one would anticipate that poorer people with lower levels of education would perceive access to care to be more problematic than those with higher education and greater income; however, this is not the case with this study. Findings in this study show that perceived access problems are significantly more common among college graduates and those who have higher incomes. This is important because it demonstrates that being poor does not necessarily indicate that a person perceives healthcare to be a problem. While being poor does not automatically mean respondents thought access to healthcare was a problem, it is important to note that a majority (62.0%) of respondents who stated that they did not have enough money for healthcare also perceived a problem with access to healthcare.

The effect of geography and its role in isolation has also been shown to affect access to healthcare for residents of Appalachia (Arcury et al., 2005; Huttlinger et al., 2004; Ramsbottom-Lucier, Emmett, Rich, &Wilson, 1996). Geography of the Hawkins County, especially the hills, valleys, and hollows, affect mobility within the county where poverty is an all too frequent issue (Wilson et al., 2010).

Having access to personal transportation is important in this rural community (there is no public transportation other than taxis, which do not go to the most rural areas of the county). It is worthy to note that although most respondents stated that they had no transportation issues, findings suggest those who did identify transportation difficulties also perceived access to healthcare to be a problem. Access to a vehicle for work or medical care is critical for independence in this rural area. Relying on others for transportation is likely seen as a form of needing charity, a factor that is not culturally acceptable in this region (Behringer et al., 2010).

The roles social influences and context play in an individual’s decision to seek healthcare are gaining increasing recognition as contributors to healthcare utilization (Behringer et al., 2007; Emmons, Barbeau, Gutheil, Stryker, & Stoddard, 2007; Wilson et al., 2010). Place, gender, beliefs and a number of important cultural issues, such as community, personal confidence, lack of assertiveness, trust, sense of personal privacy and pride, rejection of anything considered charity, and fatalism, play an important role in decisions to seek medical care (Behringer & Friedell, 2006; Behringer et al., 2007; Della, 2011; Hayes, 2006; Litaker, Koroukian, & Love, 2005). These factors were outside the purview of this study, but represent significant areas of interest for future studies. Given the relatively dispersed settlement pattern in the county, this study shows that geographic isolation also does not present a significant problem for accessing healthcare, with the exception of when transportation is not available.

Since access to healthcare was not defined for study respondents, it is likely that rural living and an individual’s perceived access differed depending on contextual factors, such as family history, perceptions of ill health, severity of illness, and expectations of health service availability. It is also possible that respondents were more likely to view local health services through one of the regional rural health services clinics that offer care on a sliding fee scale to be locally available and accessible to them, and, thus, do not perceive access to care to be a problem for them. Specialty services and advanced hospital services are not locally available except to those in the northern part of the county, with the exception of a few physicians who hold office visits in the county seat once or twice a month. Assessing these constructs may also shed light on why upper income individuals were more likely to perceive access to healthcare as a community problem than poor, low or middle income respondents. An important aspect for future study should include assessments of emic values and understandings that constitute access to healthcare in this and other populations.

Another factor that indicates we must look further for answers to access to healthcare issues is the fact that, among the individuals who reported having treatment for major chronic diseases and conditions, the statistics clearly showed they did not perceive the community had an access to healthcare problem. It could be that the sample population defines the “community” differently than the geo-political boundaries of the county, as is common for health planners.

## 5. Conclusions

Despite the presence of multiple factors previously shown to affect access to healthcare, the majority of respondents in this study did not perceive access to healthcare to be a problem in their community. The study demonstrated that financial factors and social determinants of health negatively affected only some, but not all, perceptions of access to healthcare. Of the twenty factors measured against perceived access to healthcare, only six factors contributed statistically to the probability of predicting that access to healthcare was a problem: gender, income, education, insurance, financial difficulties paying for healthcare, and transportation. Gender was a marginally significant predictor of perceived healthcare access problems. Paradoxically, wealthier, more educated respondents were more likely to perceive an access problem in their community than were poorer, less educated respondents. At the same time, lack of insurance, lack of enough money to pay for healthcare and those with transportation problems perceived healthcare access to be a problem.

Lowering or eliminating health disparities is a universal health goal that requires going beyond the four components of coverage, services, timeliness, and workforce. Results of this study suggest that to understand an individual’s passage through the healthcare system, the contextual aspects of healthcare utilization, including but not limited to, examination of why and how individuals seek healthcare services should be added as a fifth component of access to care. Assessing perceived need and associated cultural factors that affect individuals’ concepts of health and wellness represent important areas for future exploration to explain observed health disparities. Additionally, findings showed that having sufficient quality and quantity of healthcare professionals and services in a community or region may be necessary, but not sufficient to explain health disparities and the underlying reasons why individuals choose or choose not to seek health services.
